# P-834. Epidemiology and Duration of Therapy in Patients with Gram-negative Bloodstream Infections: Retrospective Analysis 2.15.0.02.15.0.0

**DOI:** 10.1093/ofid/ofae631.1026

**Published:** 2025-01-29

**Authors:** Hawra J Al Lawati, Ellen Earle, Matthew S Lee

**Affiliations:** Beth Israel Deaconess Medical Center, Boston, Massachusetts; Beth Israel Deaconess Medical Center, Boston, Massachusetts; Beth Israel Deaconess Medical Center, Boston, Massachusetts

## Abstract

**Background:**

Longer courses of antibiotics can be associated with antimicrobial resistance and adverse effects. Randomized clinical trials support treating uncomplicated gram-negative bloodstream infections (GN-BSI) for a shorter duration with a consensus that a seven-day course of antibiotics is appropriate. Prior to the implementation of a GN-BSI treatment guideline at our institution, we aimed to assess the characteristics of patients with GN-BSI and the duration of antibiotic therapy (DOT).

**Figure:**
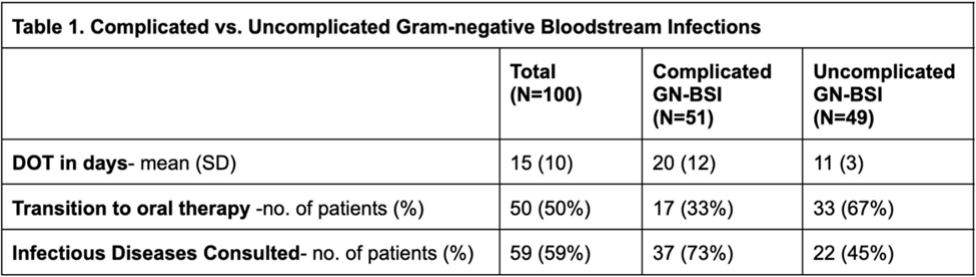

**Methods:**

We retrospectively reviewed adult inpatients with GN-BSI over a period of 6 months. Patients were excluded if they had a concomitant gram-positive bloodstream infection or if they were transitioned to comfort-focused care within 48 hours of their first positive blood culture. Complicated GN-BSI was defined as having any of the following: bone, joint, endovascular, or foreign body involvement, lack of source control, immunocompromised status, or failure to show clinical improvement or culture clearance within 72 hours. The primary outcome was the mean DOT in patients with GN-BSI.

**Results:**

100 patients met the inclusion criteria. Escherichia coli (54 cases) was most frequent organism. Urine (41) was the predominant source of bacteremia. Cefepime (48) was the most common empiric antibiotic. Of the 91 patients with available ceftriaxone susceptibility results, 84% had a susceptible organism. Of the 51 patients with a complicated GN-BSI, the leading cause was immunosuppression. Table 1 presents a comparative analysis of complicated vs. uncomplicated GN-BSI. Complicated GN-BSI had longer DOT (20 vs. 11 days, P< 0.005) and fewer patients transitioned to oral therapy (33% vs. 67%, P< 0.005).

**Conclusion:**

At our institution, patients with uncomplicated GN-BSI have a shorter DOT and are more likely to transition to oral therapy than those with complicated GN-BSI. However, the mean DOT for uncomplicated infections remained longer than seven days and a large number of uncomplicated GN-BSI patients did not transition to oral therapy, indicating room for improvement in local practice through antimicrobial stewardship initiatives.

**Disclosures:**

**All Authors**: No reported disclosures

